# Risk factors for overweight and obesity among women of reproductive age in Dar es Salaam, Tanzania

**DOI:** 10.1186/s40795-021-00445-z

**Published:** 2021-07-16

**Authors:** Dominic Mosha, Heavenlight A. Paulo, Mary Mwanyika-Sando, Innocent B. Mboya, Isabel Madzorera, Germana H. Leyna, Sia E. Msuya, Till W. Bärnighausen, Japhet Killewo, Wafaie W. Fawzi

**Affiliations:** 1 Africa Academy for Public Health, Dar es Salaam, Tanzania; 2 Ifakara Health Institute, Dar es Salaam, Tanzania; 3 Department of Epidemiology and Biostatistics, Institute of Public Health, Kilimanjaro Christian Medical University College, Moshi, Tanzania; 4 Department of Epidemiology and Biostatistics, Muhimbili University of Health and Allied Sciences, Dar es Salaam, Tanzania; 5 School of Mathematics, Statistics and Computer Science, University of KwaZulu Natal, Durban, South Africa; 6 Department of Global Health and Population, Harvard TH Chan School of Public Health, Boston, USA; 7 Tanzania Food and Nutrition Centre, Dar es Salaam, Tanzania; 8 Institute of Global Health, University of Heidelberg, Heidelberg, Germany; 9 Department of Nutrition, Harvard TH Chan School of Public Health, Boston, USA

**Keywords:** Overweight, Obesity, Women, Nutrients, Physical activity, Tanzania

## Abstract

**Background:**

Overweight and obesity have increased considerably in low- and middle-income countries over the past few decades, particularly among women of reproductive age. This study assessed the role of physical activity, nutrient intake and risk factors for overweight and obesity among women in Dar es Salaam, Tanzania.

**Methods:**

We conducted a cross-sectional survey among 1004 women aged 15–49 years in the Dar es Salaam Urban Cohort Study (DUCS) from September 2018 to January 2019. Dietary intake was assessed using a food frequency questionnaire (FFQ). Physical activity was assessed using the Global Physical Activity Questionnaire (GPAQ) using metabolic equivalent tasks (MET). Modified poison regression models were used to evaluate associations between physical activity and nutrient intake with overweight/obesity in women, controlling for energy and other factors.

**Results:**

The mean (±SD) age of study women was 30.2 (±8.1) years. Prevalence of overweight and obesity was high (50.4%), and underweight was 8.6%. The risk of overweight/obesity was higher among older women (35–49 vs 15–24 years: PR 1.59; 95% CI: 1.30–1.95); women of higher wealth status (PR 1.24; 95% CI: 1.07–1.43); and informally employed and married women. Attaining moderate to high physical activity (≥600 MET) was inversely associated with overweight/obesity (PR 0.79; 95% CI: 0.63–0.99). Dietary sugar intake (PR 1.27; 95% CI: 1.03–1.58) was associated with increased risk, and fish and poultry consumption (PR 0.78; 95% CI: 0.61–0.99) with lower risk of overweight/obesity.

**Conclusion:**

Lifestyle (low physical activity and high sugar intake), age, wealth status, informal employment and marital status were associated with increased risk of overweight/obesity, while consumption of fish and poultry protein was associated with lower risk. The study findings underscore the need to design feasible and high-impact interventions to address physical activity and healthy diets among women in Tanzania.

## Background

Overweight and obesity are major public health concerns affecting about half of the global adult population, with prevalence being greater among women compared to men [1, 2]. In low- and middle-income countries (LMICs), overweight and obesity have been increasing at a rapid rate, particularly in urban compared with rural settings [3]. Tanzania is not an exception, with overweight and obesity among women of reproductive age increasing markedly from 21.5 to 28% from 2010 to 2015 [4]. The prevalence of overweight among women in urban areas is twice (42%) as high as that for women in rural areas (21%) [5].

The effects of overweight and obesity on health are well researched. The global burden of disease related to high body mass index (BMI) is high. Globally, high BMI accounts for more than 4 million deaths, two-thirds of which are due to cardiovascular disease [6]. In LMICs, the disability-adjusted life years (DALYs) related to obesity are high and have been steadily rising compared to high-income countries [7]. Among women of childbearing age, overweight and obesity have been associated with increased risk of non-communicable diseases (NCDs), pregnancy complications, caesarean section births, adverse birth outcomes, and infant mortality [8–11]. A recent study conducted in Tanzania reported associations between maternal overweight and increased risk of intrapartum obstetric complications and caesarean section births [12]. Furthermore, offspring of obese mothers have had up to 29% increased risk of hospital admission from cardiovascular disease and 35% increased risk of premature death in adulthood compared to offspring of normal BMI mothers [13].

Several factors may be contributing to increasing overweight and obesity among women in low-income settings and sub-Saharan Africa (SSA), and these include environmental and lifestyle factors, genetics and diseases [14]. Additionally, high socioeconomic status, increasing age, parity, and marital status have also been associated with overweight among women in the region [15, 16].

Physical inactivity and poor dietary patterns characterised by high intake of calorie-rich, processed and refined foods may be key modifiable risk factors for overweight and obesity in SSA [15, 16]. However, little is known about the role of physical activity and nutrients intake among African women of reproductive age because most reports come from national demographic surveys where physical activity and nutrient intake are not assessed. Previous studies in urban African settings assessing nutrient intake among women of reproductive age are limited by small sample sizes affecting the generalisation of their findings [17, 18]. Additionally, understanding the actual contribution of physical activity to women’s BMI is a bit tricky considering women’s participation in energy-demanding domestic activities to support household needs, which is often unaccounted. A clear understanding of the role of these modifiable risk factors in overweight and obesity in the African context may assist in designing appropriate interventions given unprecedented urbanisation, nutrition and dietary transition observed in many African cities [19, 20].

High prevalence of environmental and lifestyle diseases related to high BMI cannot be ignored in Tanzania, considering the upsurge of maternal BMI in urban settings recently reported from the Tanzania national Nutrition Survey [21]. As a country, no effective policies and programs to control overweight in women of reproductive age. This may be due to several factors, including limited pre-pregnancy BMI data from clearly designed populations studies in Tanzania, as in other low-income settings. This study aimed to determine the prevalence and factors associated with overweight and obesity and evaluate the role of physical activity in high BMI among women of reproductive age in Dar es Salaam, Tanzania.

## Methods

The study was conducted in the Dar es Salaam Urban Cohort Study (DUCS) site, a Health and Demographic Surveillance System (HDSS) platform based in Ukonga and Gongolamboto wards, in Ilala District of Dar es salaam. The platform is a peri-urban area in Dar es Salaam, the commercial city of Tanzania. The design of the DUCS and the study population have been described in detail elsewhere [22].

The study was a cross-sectional study nested in the HDSS platform. We enrolled women of reproductive age from September 2018 to January 2019. A list of women of reproductive age was initially pulled from the HDSS database that contained participants’ names, dates of birth, and household identification numbers. A simple random sampling technique using random numbers was applied to select households with women of reproductive age. A field worker then visited the selected households to identify women who met the inclusion criteria. Inclusion criteria for the study included (i) women aged 15 to 49 years, (ii) who intended to become pregnant within the next 4 years, (iii) were currently not pregnant based on the last normal menstrual period, and (iv) provided written informed consent. A lottery method was used to select one woman randomly from households with more than one woman. A household replacement was considered for those households in which no woman met the inclusion criteria.

The sample size for the study was calculated based on multiple indicators using a cluster survey formula that factored in the predicted prevalence of overweight/obesity (42%), the proportion of women of reproductive age (30%), average household size in Dar es Salaam (4.0), and the anticipated non-response [5]. Therefore, the minimum sample size was 1012 women for 80% study power and less than 5% level of significance.

Face-to-face interviews were conducted for data collection using a standardized questionnaire. Study research assistants collected information on participants’ socio-demographic and economic characteristics, lifestyle characteristics such as alcohol use, smoking, dietary intake, and levels of physical. Information on pregnancy status and medical history was also collected. Anthropometric measurements, including weight and height, were taken using a calibrated weighing scale to the nearest 100 g and a height board to the nearest cm.

### Outcome variable

The outcome in this study was overweight and obesity obtained by computing BMI as weight in kilograms (Kg) divided by height in meters (m) squared. BMI categories followed the WHO recommendations as < 18.50 kg/m^2^ (underweight), 18.50–24.99 kg/m^2^ (normal), 25–29.9 kg/ m^2^ (overweight) and ≥ 30 kg/m^2^ (obese) [2]. A binary outcome variable was generated by combining overweight or obese and compared against women who had normal BMI.

### Assessment of physical activity

Research assistants collected information on physical activity using the Global Physical Activity Questionnaire (GPAQ) [23]. Metabolic Equivalents (MET) assessed physical activity levels [23]. The value of MET for each reported physical activity for each woman was obtained from the compendium of physical activity types. Physical activity was categorized into two groups: moderate-intensity and vigorous-intensity. Moderate-intensity physical activity included brisk walking, dancing, housework and domestic chores, gardening, animal rearing**,** washing clothes, fetching water, and preparing food. Vigorous-intensity physical activity included climbing a hill, running, fast cycling, swimming, intense farming, competitive sports, traditional games, and wood splitting for fire. Total physical activity (MET-minutes per week) was calculated based on analysis guidelines for physical activity recommended by WHO [24]. Moderate to vigorous physical activity was scored as ≥600 MET-minutes per week, while sedentary physical activity was scored as < 600 MET-minutes per week. Total time spent in vigorous physical activity was categorized as ≥75 min per week and < 75 min per week, and moderate physical activity was categorized as ≥150 min per week and < 150 min per week.

### Assessment of macronutrients intake

Dietary information was assessed using a locally adapted Food Frequency Questionnaire (FFQ) used previously in the study area, containing at least 85 foods [25]. Women were asked to recall foods consumed in the previous 30 days. Nutrient intake was assessed from each reported food consumed by the respondent, based on the Tanzania Food Composition Table (TFCT) [26]. Nutrient values of each food item were calculated by multiplying each food item’s frequency of consumption by the food item’s nutrient content and the specific portion size. Nutrient intake was categorized into tertiles, namely low, medium, and high intake tertiles.

#### Statistical analysis

Data were cleaned and analyzed by using STATA version 15. Numerical variables were summarized using means and standard deviations, and medians and interquartile range. Categorical variables were summarized using frequencies and percentages. We used the chi-square test to compare the proportion of women with overweight and obesity across explanatory variables, including social-demographic characteristics, physical activity, and dietary diversity.

Potential confounders for each outcome were selected based on associations with the outcome in bivariate regression models at levels of *p* < 0.1. Confounders considered included age, marital status, education, parity, wealth index, and employment type. The final model was adjusted by total energy intake. Please note, wealth index was created using principal component analysis then categorised into tertile.

A modified Poisson regression model with a robust standard error estimated prevalence ratios (PR) and 95% confidence intervals (CI) [27]. The model was used to evaluate the association between physical activity and nutrient intake on overweight and obesity. Modified Poisson regression was used due to the non-convergence of the log-binomial regression model [27]. All analyses were based on a two-tailed significance level at *p* < 0.05. The Akaike Information Criteria (AIC) was used for model selection, whereby the model with the lowest AIC was considered as a parsimonious model. Tests for trend were conducted for multivariate models for macronutrients.

## Results

A total of 1004 women of reproductive age were enrolled in the study. Women in the study had a mean age (±SD) of 30.2 (±8.1) years. Of these, 31.7% were 35 years or older, 57.9% were either married or cohabiting, and 54.4% had no employment. Fifty-four percent of the women had at least two children. The nutrition status of study women was poor, with 8.6% underweight, 27.8% overweight, and 22.6% obese. The overall/combined prevalence of overweight and obesity was 50.4% [Table 1].

**Table 1:** Characteristics of women enrolled in the study (*N* = 1004)

Variable	Frequency	Percentage
**Age group (years)**		
15–24	334	33.3
25–34	351	35.0
35 and above	319	31.7
**Education level**		
No education	59	5.9
Primary	600	59.8
Secondary	267	26.6
Above secondary education	78	7.7
**Employment**		
No employment	546	54.4
Informal employment	334	33.3
Formal employment	124	12.4
**Marital Status**		
Single	356	35.5
Married/cohabiting	581	57.9
Divorced/separated/widow	66	6.6
**Parity**		
None	267	26.6
One	194	19.3
Two and above	543	54.1
**Household size**		
1–3	170	16.9
4–5	333	33.2
6 and above	501	49.9
**BMI (Kg/M**^**2**^**)**		
^**a**^Mean (±SD)	25.8	±5.8
Underweight	86	8.6
Normal	412	41.0
Overweight	279	27.8
Obese	227	22.6

^**a**^Mean (±Standard deviation)

**Fig. 1:**
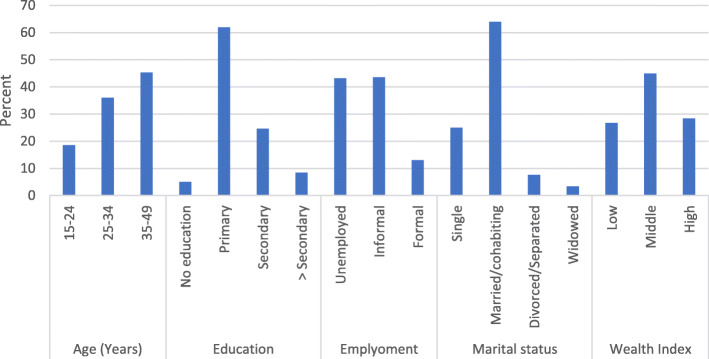
Socio-demographic characteristics of overweight and obese women with sufficient physical activity (*N* = 220)

About 43 % (220/506) of overweight and obese women had total physical activity above 600 metabolic equivalents of task (MET for moderate to vigorous physical activity), referred to as sufficient physical activity. Among the overweight and obese women with sufficient total physical activity, 61.9% had primary education level, and 64.0% were married or cohabiting. Additionally, 3.4% of the women were widowed, 5.1% uneducated, and 8.5% with an education level above secondary school [Fig. 1].

Results from the adjusted analysis for factors associated with overweight and obesity are in Table 2. Compared to women aged 15–24 years, women aged 25–34 years had a 26% higher risk of overweight and obesity (95%CI 1.03–1.54; *p* = 0.03), while those aged 35 to49 years had a 59% higher risk of the outcome (95%CI 1.30–1.95; *p* < 0.001), adjusted for physical activity and energy intake. Women who had informal employment had a 14% higher risk of overweight and obesity (95% CI: 1.01–1.29; *p* = 0.04) compared with those who are not employed. Married or cohabiting women had a 33% higher risk of overweight and obesity (95% CI: 1.11–1.60; *p* < 0.01) compared with single, divorced or separated women after adjusting for physical activity and energy intake. However, other factors, including education, parity and household size, were not significantly associated with overweight or obesity. Women in the medium and high wealth index tertiles had 25% (PR = 1.25; 95%CI 1.09–1.43; p < 0.01) and 24% (PR = 1.24; 95%CI 1.07–1.43; p < 0.01) higher prevalence of overweight and obesity, compared to women in the low wealth index tertiles in models adjusting for physical activity and energy intake [Table 2].

**Table 2:** Associations between participant socio-demographic characteristics and overweight/obesity among women of reproductive age in Dar es Salaam, Tanzania (*N* = 918)

Variables	N	n (%)	CPR(95%CI)	***P***-value	APR^**a**^(95%CI)	***P***-value
**Age in years**						
15–24	334	105 (31.4)	1		1	
25–34	351	185 (52.7)	1.50 (1.25,1.79)	< 0.01	1.26 (1.03,1.54)	0.03
35–49	319	218 (68.3)	1.90 (1.61,2.45)	< 0.01	1.59 (1.30,1.95)	< 0.01
**Household size**						
1–3	170	88 (51.8)	1			
4–5	333	181 (54.4)	1.09 (0.84,1.41)	0.52		
6 and above	501	239 (47.7)	0.94 (0.73,1.21)	0.64		
**Education**						
No education	59	28 (47.5)	1			
Primary	600	329 (54.8)	1.12 (0.86,1.16)	0.40		
Secondary	267	114 (42.7)	0.94 (0.70,1.25)	0.67		
Above secondary	78	37 (47.4)	1.04 (0.75,1.47)	0.78		
**Type of Employment**						
No employment	546	242 (44.3)	1		1	
Informal	334	199 (59.6)	1.28 (1.13,1.45)	< 0.01	1.14 (1.01,1.29)	0.04
Formal	124	67 (54.0)	1.17 (0.98,1.40)	0.08	1.06 (0.90,1.26)	0.48
**Marital Status**						
Single	356	114 (32.0)	1		1	
Married/cohabiting	581	357 (61.5)	1.66 (1.42,1.94)	< 0.01	1.33 (1.11,1.60)	< 0.01
Divorced/separated	66	36 (54.6)	1.52 (1.17,1.97)	< 0.01	1.21 (0.93,1.59)	0.16
**Parity**						
0–1	194	97 (50.0)	1			
> 1	543	329 (60.6)	1.16 (1.00,1.34)	0.06		
**Wealth index Score**						
Low	335	150 (44.8)	1		1	
Middle	395	210 (53.2)	1.21 (1.05,1.39)	0.01	1.25 (1.09,1.4)	< 0.01
High	274	148 (54.0)	1.18 (1.12,1.61)	0.03	1.24 (1.07,1.4)	< 0.01

*Abbreviations*: *CPR* Crude prevalence ratio; *APR* Adjusted prevalence ratio

^a^Adjusted for physical activity and energy intake

Total physical activity and minutes per week of vigorous physical activity were inversely associated with overweight and obesity adjusted for other factors [Table 3]. Women who performed moderate to vigorous physical activity of at least 600 MET per week had a 21% lower prevalence of overweight and obesity compared with those with a sedentary lifestyle (PR = 0.79; 95% CI 0.63–0.99; *p* = 0.04). Women who performed vigorous physical activity at least 75 min per week had a 32% lower prevalence of overweight and obesity than those who performed less than 75 min of vigorous physical activity per week (PR = 0.68; 95%CI 0.47–0.99; p = 0.04). However, work-related moderate physical activity (minutes per week of moderate physical activity) and days of moderate physical activity were not significantly associated with overweight and obesity [Table 3].

**Table 3:** Associations of physical activity and the risk of overweight and obesity among women of reproductive age in Dar es Salaam, Tanzania (n = 918)

Variable	CPR(95%CI)	***P***-value	APR ^a^(95%CI)	***P***-value
**Total physical activity**				
Sedentary (≤600 MET)	1		1	
MVPA (> 600 MET)	1.00 (0.89,1.13)	0.99	0.79 (0.63,0.99)	0.04
**Minutes per week of vigorous physical activity**				
< 75 min	1		1	
≥ 75 min	1.13 (0.86,1.48)	0.39	0.68 (0.47,0.99)	0.04
**Minutes per week of moderate physical activity**				
< 150 min	1		1	
≥ 150 min	1.01 (0.88,1.15)	0.93	1.02 (0.81,1.27)	0.90
**Days of vigorous physical activity per week**				
None	1			
1–3 days/week	1.32 (1.03,1.71)	0.03		
4–7 days/week	1.15 (0.92,1.45)	0.22		
**Days of moderate physical activity per week**				
None	1			
1–3 days/week	0.91 (0.73,1.14)	0.43		
4–7 days/week	1.01 (0.89, 0.14)	0.92		
**Days of walking or driving a bicycle at least 10 min**				
None	1			
1–3 days/week	0.90 (0.78, 1.04)	0.15		
4–7 days/week	0.97 (0.84, 1.11)	0.67		

*Abbreviations*: *CRP* Crude prevalence ratio; *APR* Adjusted prevalence ratio; *MET* Metabolic Equivalent of Task; *MVPA* Moderate to Vigorous Physical Activity

^a^Adjusted prevalence ratio – adjusted for energy intake, age, wealth index, type of employment, marital status, education and number of parity

Higher intake of sugar, total fat, animal protein were positively associated with overweight and obesity. In comparison, a higher intake of protein from fish and poultry was associated with a lower risk of overweight and obesity. There was a significant increase in overweight and obesity with the increased consumption of animal protein. Women in the highest tertile of animal protein intake had a higher risk of overweight and obesity (PR = 1.19, 95%CI: 1.01, 1.35), p for trend 0.26. Similarly, women in the highest tertile of sugar consumption had a higher risk of overweight/obesity (PR = 1.27, 95%CI: 1.03, 1.58, p for trend < 0.01). Women in the highest tertile of protein intake from fish and poultry meat had a 22% lower risk of overweight and obesity (PR = 0.78, 95%CI: 0.61.0.99, p for trend 0.03) [Table 4].

**Table 4:** Associations between Macronutrient Intake with overweight/obese among women of reproductive age in Dar es Salaam, Tanzania (N = 918)

Macronutrients	Overweight /obese n(%)	CPR ^a^(95%CI)	***P***-value	APR^a^(95%CI)	***P***-value	***P*** value for trend
**Carbohydrate (g)**						
Low	159 (52.0)	1		1		
Medium	179 (58.5)	1.07 (0.92,1.23)	0.37	1.15 (0.91,1.46)	0.25	0.47
High	168 (54.9)	1.11 (0.95,1.30)	0.19	1.15 (0.84,1.58)	0.39	
**Animal Protein(g)**						
Low	161 (52.6)	1		1		
Medium	171 (55.7)	1.13 (0.98,1.30)	0.09	1.17 (1.01,1.35)	0.03	0.26
High	168 (57.1)	1.12 (0.94,1.32)	0.20	1.19 (1.02,1.39)	0.03	
**Total fat (g)**						
Low	176 (57.5)	1		1		
Medium	176 (57.5)	1.12 (0.96,1.30)	0.14	1.21 (0.98,1.50)	0.10	0.07
High	154 (57.5)	1.13 (0.98,1.32)	0.10	1.22 (1.03,1.45)	0.02	
**Fish & Poultry protein (g)**						
Low	156 (50.5)	1		1		
Medium	174 (56.0)	1.05 (0.85,1.30)	0.63	0.83 (0.70,0.99)	0.04	0.03
High	176 (59.1)	1.04 (0.84, 1.29)	0.74	0.78 (0.61,0.99)	0.04	
**Sugar (g)**						
Low	165 (53.9)	1		1		
Medium	169 (55.2)	1.01 (0.87,1.16)	0.10	1.13 (0.98,1.31)	0.09	< 0.01
High	172 (56.2)	1.05 (0.90,1.23)	0.07	1.27 (1.03,1.58)	0.01	
**Fiber (g)**						
Low	243 (53.9)	1		1		
Medium	140 (56.7)	0.94 (0.81, 1.09)	0.42	0.95 (0.81,1.12)	0.57	0.57
High	123 (55.9)	1.02 (0.89, 1.17)	0.81	0.95 (0.77,1.17)	0.65	

*Abbreviations*: *CRP* Crude prevalence ratio

^a^Adjusted prevalence ratio -adjusted for physical activity, energy intake, age, wealth index, type of employment, marital status, number of parity and education level

## Discussion

Our findings show that the combined prevalence of overweight and obesity among women intending to become pregnant within the next 4 years is very high in Tanzania. Overall, we found that being older, having informal employment and middle to high socioeconomic status were associated with overweight and obesity among women in Dar es Salaam, Tanzania. The study found an association between vigorous physical activity and decreased overall prevalence of overweight and obesity. Sugary dietary intake was associated with an increased prevalence of overweight and obesity. Consumption of protein from fish and poultry was associated with a lower risk of overweight and obesity after adjusting for energy intake and physical activity.

More than half of the women in our study are either overweight or obese, which is higher than the 2015 national prevalence in urban settings (50.7% vs 42.0%) in Tanzania [5]. The high prevalence of overweight and obesity in the study area may explain the impact of economic development, nutrition transition and women’s empowerment on overweight and obesity that is more pronounced in African cities like Dar es salaam [17, 19]. Women in this study had a 6-fold higher prevalence of overweight and obesity when compared with the underweight (50.4% vs 8.6%). This is of great concern given most nutritional counselling observed in many antenatal health care services; the emphasis is on maternal weight gain and less on the overweight and obese control (a *personal conversation with the health facility providers in the study area*).

A high prevalence of maternal obesity is also reported in a systematic review and meta-analysis across Africa, ranging from 6.5 to 50.7% [28]. Economic and nutrition transition remain the core reasons demonstrated to expose women to sedentary life and unhealthy diets [29, 30]. Such a high prevalence of overweight and obesity in women who intend to conceive within the next few years is alarming considering the reported maternal and newborn adverse outcomes associated with high pre-pregnancy BMI [9, 11].

Being older, having informal employment, and middle to high socioeconomic status were associated with an increased prevalence of overweight and obesity in this study. These findings are consistent with other studies that reported a higher prevalence of overweight and obesity in older women [15, 31, 32]. Increased parity, hormonal changes, and a less active lifestyle may attribute obesity among older women [33, 34]. In addition, weight retained during pregnancy is often difficult for women to lose, even for obese women, contributing to increased BMI over time [35].

Women who were self-employed or under the informal employment sector, such as street vendors, shopkeepers, and tailors, had a higher prevalence of overweight and obesity than women who were unemployed or formally employed. The role of employment status as a determinant of BMI is not clear. Studies have shown that white-collar workers are at the greatest risk of low occupational physical activity levels and sedentary behaviour [36, 37]. In this study, women under formal employment in most cases were in the white-collar job category; however, this was not associated with an increased risk of overweight and obesity. There is, therefore, a need to understand the nature and actual contribution of specific employment status to women’s BMI.

We found that women with higher economic status had a higher prevalence of overweight and obesity. Similar findings have also been reported by other studies from SSA, where socioeconomic status was an important determinant of overweight and obesity [15, 32, 38]. This may be due to socio-cultural factors and perceptions in many LMICs favouring women having larger body size [39, 40]. Being obese or overweight in many African countries have been perceived as a sign of being wealthy, having enough to eat, and less associated with diseases such as HIV infection [41, 42]. More importantly, more affluent households can afford more calories in their diets, having financial power to purchase processed and unhealthy foods, eat fast food from restaurants etc., while also being less likely to be physically active [43]. The findings are contrary to many studies in high-income countries where adults with higher socioeconomic status have a low prevalence of obesity [44, 45].

Besides controlling dietary intake, having sufficient exercise and physical activity is considered an effective approach for controlling weight gain [46]. This is in line with our findings that women who met moderate to vigorous total physical activity criteria (MVPA) had a lower prevalence of overweight and obesity by 21%. Similar findings have been reported from Ghana, where women who did not meet the recommended physical activity level had an increased risk of obesity by 23% [47]. Physical activity, including aerobic exercises, reduces fat mass and body weight [48].

We found that high sugar consumption was associated with a higher prevalence of overweight and obesity, which is consistent with findings from a systematic review in SSA. The review found that a steady increase in the availability and consumption of energy-rich foods from the 1980s had contributed substantially to the increase of obesity in the region [49]. High sugar and beverages consumption above 10% of the total daily energy requirement has increased in recent years, especially in urban settings, including Tanzania [50]. Thus, this calls for immediate attention, given that high sugar intake is associated with non-communicable diseases [51]. Animal protein and fat intake were not associated with an increased risk of overweight and obesity. This is contrary to the USA and European study, which associated animal protein intake with increased global and abdominal obesity risk. Fish and poultry protein intake was significant associated with a low risk of overweight and obesity. Compared to animal (red) meat, fish and chicken have less saturated fat and cholesterol, which justifies having less risk of overweight and obesity [52].

Our study is one of the few studies in SSA that has measured physical activity and dietary intake given the current situation of unprecedented urbanization and dietary transmission in many African countries. Therefore, we believe the findings are vital to underpin the importance of addressing overweight and obesity determinants in the region, including physical activity and healthy diets. However, we cannot ignore the possibility of a recall bias as some respondents may fail to remember foods consumed in the past 30 days. Additionally, we utilize a cross-sectional study design which may be affected by confounding. However, we tried to address the confounding effect by adjusted for energy intake and known potential confounders. Additionally, in the models for dietary intake, we controlled for physical activity.

## Conclusion

The overall prevalence of overweight and obesity among women of reproductive age who intend to conceive within the next 4 years was very high. Overweight and obesity were significantly associated with a sedentary lifestyle, wealth, older age, informal employment status, and marital status. High sugar intake was associated with a higher risk of overweight and obesity, while protein consumption from fish and poultry was associated with lower risk. The findings of this study underscore the need to design culturally-sensitive, feasible and potentially high-impact interventions to address the modifiable risk factors of physical activity and healthy diets to control the upsurge of pre-pregnancy overweight and obesity among women in SSA countries.

### Availability of data and materials

The dataset generated during the current study are not publicly available due to the Africa Academy for Public Health (AAPH) data policy but are available from the corresponding author on reasonable request.

## Data Availability

The dataset of the current study is available from the corresponding author based on a reasonable request.

## Abbreviations

BMI: Body Mass Index
DUCS: Dar es Salaam Urban Cohort Study
DAILYs: Disability-Adjusted Life Years
FFQ: Food Frequency Questionnaire
GPAQ: Global Physical Activity Questionnaire
LMICs: Low- and Middle-Income Countries
MET: Metabolic Equivalent Tasks
NCD: Non-Communicable Diseases
TFNC: Tanzania Food Composition Table
WHO: World Health Organization
